# Absorption and Metabolism Characteristics of Triptolide as Determined by a Sensitive and Reliable LC-MS/MS Method

**DOI:** 10.3390/molecules20058928

**Published:** 2015-05-18

**Authors:** Xiaomei Gong, Yan Chen, Yi Wu

**Affiliations:** 1 Department of Radiatin Oncology, Shanghai Pulmonary Hospital, Tongji University, 507 Zhengmin Road, Shanghai 200433, China; 2 Department of Gastroenterology, Changhai Hospital, Second Military Medical University, 168 Changhai Road, Shanghai 200433, China; E-Mail: medchenyan@126.com; 3 Institute of Traditional Chinese Veterinary Medicine, College of Veterinary Medicine, Nanjing Agricultural University, 1# Weigang, Nanjing 210095, Jiangsu, China

**Keywords:** Caco-2 cells, human liver microsome, *P-gp*, pharmacokinetics, triptolide

## Abstract

In this research, a sensitive and reliable LC-MS/MS method was developed and applied to determine the concentration of triptolide in rat plasma, microsomes, and cell incubation media. The absolute oral bioavailability of triptolide is 63.9% at a dose of 1 mg·kg^−1^. *In vitro*, the bidirectional transport of triptolide across Caco-2 cells was studied. A markedly higher transport of triptolide across Caco-2 cells was observed in the basolateral-to-apical direction and was abrogated in the presence of the *P-gp* inhibitor, verapamil. The result indicated that *P-gp* might be involved in the absorption of triptolide in intestinal. The metabolic stability was also investigated using human liver microsome incubation systems *in vitro*. In HLMs, incubations with an initial triptolide concentration of 1 μM resulted in an 82.4% loss of substrate over 60 min, and the *t_1/2_* was 38 min, which indicated that triptolide was easily metabolized in human liver microsomes. In conclusion, the absolute oral bioavailability of triptolide in plasma, transport across Caco-2 cell monolayers, and metabolic stability in human liver microsomes were systematically investigated by using a sensitive and reliable LC-MS/MS method.

## 1. Introduction

Triptolide, a diterpenoid triepoxide, is a major pharmacological component isolated from *Tripterygium wilfordii Hook* F (TWHF) [1], and it has been used primarily for the treatment of inflammatory and autoimmune diseases such as rheumatoid arthritis, systemic lupus erythematosus, and skin diseases [2,3]. Recently, several researches have reported that triptolide possess potent anti-cancer effect, such as gastric cancer, pancreatic cancer, and lung cancer [4,5,6]. Therefore, triptolide has the potential of becoming a leading compound of anti-cancer drug. However, the clinical application of triptolide was restricted because of its narrow therapeutic range and severe toxicity to digestive, reproductive and hematopoietic systems [2,7].

As we know, triptolide has been used in the clinic to treat some diseases. Therefore, to enhance the development potentials of triptolide as a chemotherapeutic agent and avoid adverse drug-drug interactions, there is a great need to further understand the *in vitro* and *in vivo* absorption and metabolism characteristics of triptolide. Several papers have focused on the pharmacokinetic or phase-I metabolism of triptolide alone [8,9,10,11], however, there has been little research concerning its absolute oral bioavailability, intestinal absorption and metabolism mechanism(s). Furthermore, several analytical methods have been developed for the determination of triptolide alone or with other components, but methods that determine triptolide in different matrixes were not found in the published literature [12,13,14,15,16,17]. The aim of this study was to investigate its pharmacokinetic characteristics after oral or intravenous administration of triptolide by using a sensitive and specific LC-MS/MS method, and then further clarify the mechanisms of absorption by Caco-2 cell monolayer model, and metabolic stability using human liver microsome incubation systems *in vitro*.

## 2. Results and Discussion

### 2.1. Chromatography and Mass Spectrometry

**Figure 1:**
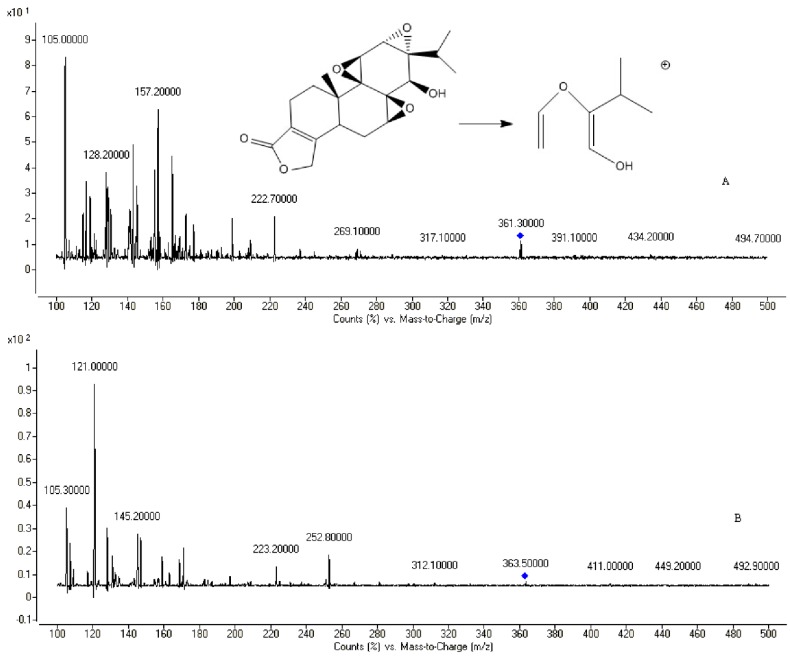
The mass spectra of triptolide (**A**) and IS (**B**).

A sensitive and reliable method to determine the concentration of triptolide had been established. Acetonitrile was used to directly precipitate plasma proteins, which could improve the recovery of triptolide. This newly established method enabled us to determine the concentration of triptolide *in vivo* after administration of a low dose of triptolide, which is moderately toxic. Representative MRM chromatograms and the structures of triptolide and the IS are shown in Figure 1, along with the optimized mass transition ion-pairs for quantification, including precursor and product ions, *m/z* 361.3→128.2 for triptolide and *m/z* 363.5→121.0 for IS, respectively.

**Figure 2:**
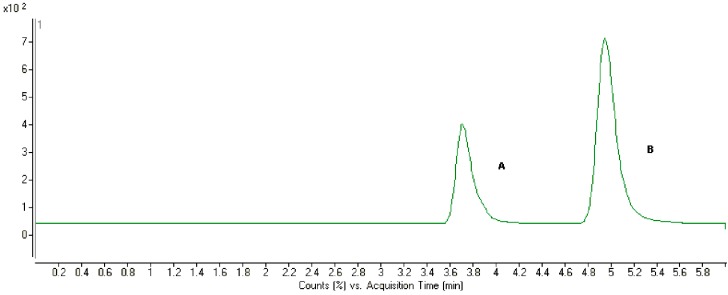
Chromatograms of plasma spiked with triptolide and IS. A: IS; B: triptolide.

Plasma spiked with triptolide and hydrocortisone are shown in Figure 2. No significant interfering substances were observed at the retention time of triptolide in plasma samples. This method also allowed us to determine triptolide concentrations in microsomes or cell transport media samples.

### 2.2. Method Development

After comparing the matrices in different media, we found that plasma is the most complex matrix among all the samples (Caco-2 cell transport media, microsomes) collected from various studies, and therefore, the method validation was conducted in rat plasma. The standard curve for triptolide in plasma was linear in the concentration range of 5–1000 ng·mL^−1^ with correlation coefficient values > 0.996. The LLOQ and LLOD were 1.72 ng·mL^−1^ and 0.59 ng·mL^−1^, respectively. The standard curves for triptolide in other matrix were all linear in the concentration range of 5–1000 ng·mL^−1^ with correlation coefficient values > 0.99. Intra-day and inter-day precision and accuracy were determined by measuring six replicates of QC samples at three concentration levels in rat plasma. The precision and accuracy data are shown in Table 1. These results demonstrated that the precision and accuracy values were well within an acceptable range of 15%. The precision and accuracy in other matrix were all within an acceptable range of 15%.

**Table 1:** The precision and accuracy of triptolide in plasma samples.

Analyte	Plasma Samples (ng/mL)	Intra-Day			Inter-Day		
		Concentration measured (ng/mL)	Precision (%, RSD)	Accuracy (%, RE)	Concentration measured (ng/mL)	Precision (%, RSD)	Accuracy (%, RE)
Triptolide	10	10.58	5.4	5.80	9.23	8.2	−7.70
	50	46.35	4.8	−7.30	54.35	7.5	8.70
	500	441.54	2.3	−11.69	551.24	4.5	10.24

The mean extraction recoveries determined using three replicates of QC samples at three concentration levels in rat plasma were 87.6% ± 4.9%, 86.8% ± 3.1%, and 91.3% ± 4.5% for 10, 50 and 500 ng·mL^−1^, respectively. The effects of the plasma matrix on ionization efficiency were evaluated by comparing the mean peak area of the supernatant spiked after the processed samples from five different lots of plasma to the pure standards at three concentrations. The matrix effect ranging from 92.6% to 107.5%, and all of the percentages were within 100% ± 15%, which meant that no significant co-eluting endogenous substances interfered with the ionization of the analytes. The Caco-2 cell transport media and microsome samples also had no significant matrix effect after comparing the standard curve of these two matrices with their respective aqueous samples. 

The stability of triptolide in plasma was evaluated by analyzing three replicates of quality control samples containing 10, 50 and 500 ng·mL^−1^ triptolide after short-term storage (25 °C, 24 h), long-term cold storage (−40 °C, 14 days) and within three freeze-thaw cycles at −40 °C. As shown in Table 2, all of the samples displayed 90%–110% recoveries after the various stability tests. Taken together, the above results show that a sensitive and reliable method for analyzing triptolide in multiple sample matrixes has been developed and validated and can be used to analyze triptolide in plasma, microsome, and Caco-2 transport media.

**Table 2:** Stability of triptolide in plasma samples (n = 3).

Analyte	Plasma Samples (ng/mL)	Stability (%, RE)		
		Short-Term (24 h at room temperature)	Long-Term (30 days at −40 °C)	Three Freeze-Thaw Cycles at −40 °C
Triptolide	10	8.1	3.8	−4.1
	50	−7.5	7.4	6.8
	500	9.98	8.5	9.54

### 2.3. Pharmacokinetic Studies

**Figure 3:**
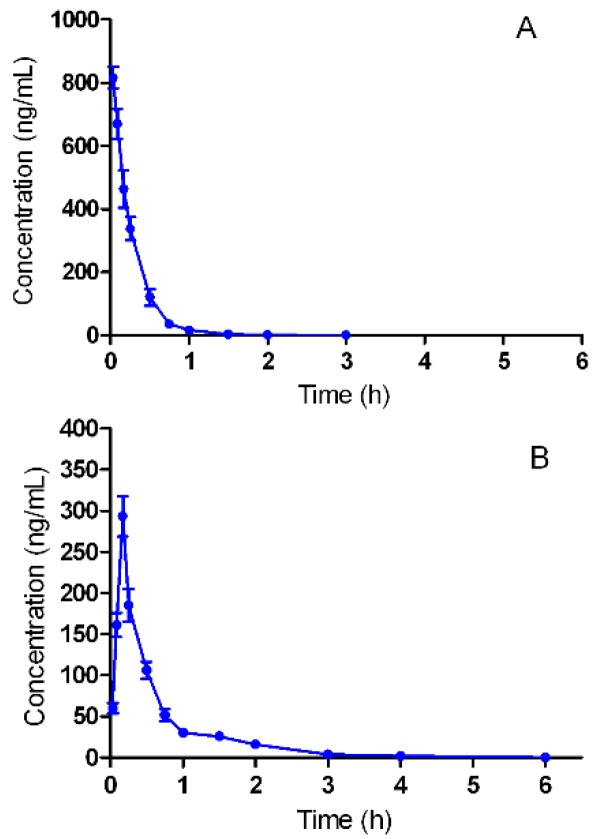
The pharmacokinetic profiles of triptolide in rats after of intravenous administration of triptolide (**A**) at a dosage of 1 mg/kg and oral administration of triptolide (**B**) at 1 mg/kg.

The validated analytical method was employed to study the pharmacokinetic behavior of triptolide in rats. The mean plasma concentration-time curves of triptolide after intravenous and oral administration are presented in Figure 3.

The pharmacokinetic parameters were calculated using the noncompartmental method with DAS 3.0 pharmacokinetic software (Chinese Pharmacological Association, Anhui, China). The pharmacokinetic parameters are shown in Table 3. The oral bioavailability was calculated by using *AUC_oral/dose_* divided by *AUC_iv/dose_*.

After 1 mg·kg^−1^ intravenous injection, triptolide concentration reached a maximum of 816.19 ± 34.44 ng·mL^−1^ and then declined rapidly. After oral administration of the same dose, triptolide was readily absorbed and reached *C_max_* 293.19 ± 24.43 ng·mL^−1^ at approximately 10 min. The *t_1/2_* was 0.42 h, which indicated that triptolide can be eliminated quickly after oral administration. For a drug administrated by oral route, oral bioavailability is one of the most important pharmacokinetics parameters. The absolute bioavailability of triptolide by oral route was 63.9%. Shao *et al.* have reported that the oral absolute bioavailability in rats was 72.08% at the dose of 0.6 mg/kg. The results indicated that the oral bioavailability of triptolide was good. 

**Table 3:** Pharmacokinetic parameters of triptolide in rats after oral or intravenous administration of triptolide (1 mg·kg^−1^; n = 6, Mean ± S.D.).

Parameter	Oral	Intravenous
Tmax (h)	0.17 ± 0.03	-
Cmax (μg·L^−1^)	293.19 ± 24.43	-
*t_1/2_* (h)	0.42 ± 0.23	0.19 ± 0.01
AUC (0-t) (μg·h·L^−1^)	151.16 ± 17.69	236.50 ± 26.40
AUMC (0-t) (μg·h·L^−1^)	112.99 ± 21.20	57.35 ± 9.76
CL (L·h^−1^·kg^−1^)	6.67 ± 0.78	4.26 ± 0.48
MRT (h)	0.74 ± 0.05	0.24 ± 0.02

### 2.4. Transport of Triptolide across a Caco-2 Cell Monolayer Model

To investigate the absorption mechanism of triptolide, a Caco-2 cell monolayer model was used. The Caco-2 cell model is an important *in vitro* model for evaluating intestinal absorption of drug. Transport of 20 μM triptolide across the Caco-2 cell monolayer model was investigated at pH 7.4. The *P_appAB_* and *P_appBA_* values were 1.34 ± 0.31 × 10^−5^ cm·s^−1^ and 2.97 ± 0.56 × 10^−5^ cm·s^−1^, respectively. The results indicated that the absorption of triptolide was good. However, The *P_appBA_* was much higher than *P_appAB_*, which indicated that efflux transporters might be involved in the transport of triptolide. 

**Figure 4:**
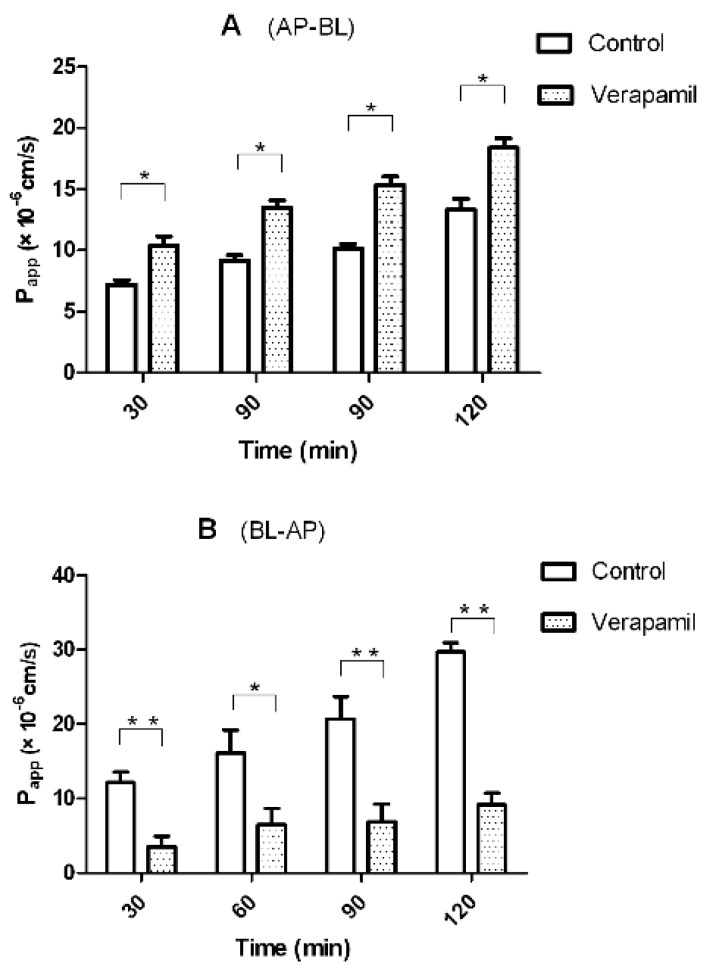
Effect of verapamil on the bidirectional transport of triptolide from the apical to basolateral side (**A**) or the opposite direction (**B**), Caco-2 cell monolayers were incubated at 37 °C in HBSS (pH 7.4), and triptolide (10 μM) was added to the donor chamber of the Caco-2 cell monolayer. Verapamil (50 μM) was added to both the donor or receiver chamber. Each point represents the mean ± SD of three determinations. * *p* < 0.05 and ** *p* < 0.01 indicate significant differences compared with the control.

Transport studies were performed in the presence of *P-gp* inhibitor (verapamil) to determine the effect of *P-gp* on the transport of triptolide. If triptolide was a substrate of *P-gp*, verapamil would inhibit its efflux (from BL side to AP side) and augment its influx (from AP side to BL side). As shown in Figure 4, in the presence of 50 μM verapamil, the *P_app_* values from AP side to BL side increased, whereas those from BL side to AP side decreased. The efflux ratio decreased from 2.2 to 0.5, and the efflux was totally inhibited. These results indicated that *P-gp* was involved in the transport of triptolide. In conclusion, when triptolide was orally administrated with *P-gp* inhibitors, the plasma concentrations of triptolide should be monitored carefully to avoid the occurrence of toxicity events.

### 2.5. Metabolic Stability of Triptolide in Human Liver Microsomes

**Figure 5:**
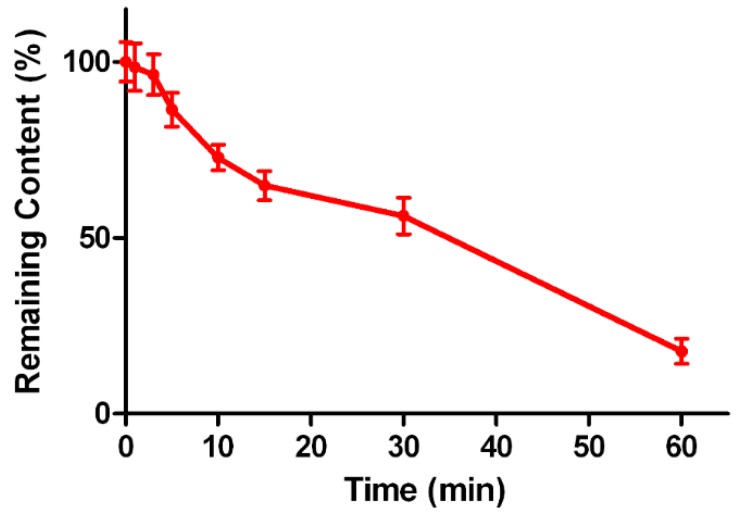
Elimination curves of triptolide after incubation in human liver microsomes *in vitro*.

*In vitro* metabolism could be used to investigate whether triptolide underwent extensive first-pass metabolism in liver after oral administration. The incubation studies were performed with human liver microsomes. The elimination curves are presented in Figure 5. In pooled human liver microsomes, incubation with an initial triptolide concentration of 1 μM caused a 82.3% ± 3.6% loss of substrate over 60 min, and the *t_1/2_* was 38 min, which indicated that triptolide could be easily metabolized by HLMs, and this results was consistent with the short *t_1/2_* values in pharmacokinetics data, that triptolide could be eliminated quickly after oral administration. 

## 3. Experimental Section

### 3.1. Chemicals and Reagents

Triptolide (purity > 98%) and hydrocortisone (purity > 98%) were purchased from the National Institute for the Control of Pharmaceutical and Biological Products (Beijing, China). Lucifer yellow, MK-571, glucose 6-phosphate (G6P), glucose 6-phosphate dehydrogenase (G6PDH), β-nicotinamide adenine dinucleotide phosphate (NADP), and tris (hydroxymethyl) aminomethane hydrochloride (Tris-HCl) were all provided by Sigma (St. Louis, MO, USA). Human liver microsome was purchased from BD GentestTM (Becton Dickinson, Franklin Lakes, NJ, USA). The Alkaline Phosphatase Assay Kit was provided by Nanjing Jiancheng Bioengineering Institute (Nanjing, China). Acetonitrile and methanol were purchased from Fisher Scientific (Fair Lawn, NJ, USA). Formic acid was purchased from Anaqua Chemicals Supply Inc. Limited (Houston, TX, USA). Ultrapure water was prepared with a Milli-Q water purification system (Millipore, Billerica, MA, USA). All other chemicals were of analytical grade or better. Dulbecco’s modified Eagle’s media (DMEM) high glucose media and non-essential amino acid (NEAA) solution were purchased from Thermo Scientific Corp (Logan, UT, USA). Fetal bovine serum (FBS) and Hanks’ balanced salt solution (HBSS) were obtained from GIBCO BRL (Grand Island, NY, US). Penicillin G (10,000 U/mL) and streptomycin (10 mg/mL) were purchased from Amresco (Solon, OH, USA). 

### 3.2. Instrumentation and Conditions

The analysis was performed on an Agilent 1290 series liquid chromatography system (Agilent Technologies, Palo Alto, CA, USA), including a binary pump, an on-line vacuum degasser, a surveyor autosampling system, a column temperature controller, and an Agilent 6460 triple-quadruple mass spectrometer (Agilent Technologies) with Turbo Ion spray, which is connected to the liquid chromatography system. The Agilent MassHunter B 4.0 software was used for the control of equipment, data acquisition, and Agilent Quantitative analysis software was used for data analysis. The chromatographic analysis of triptolide was performed on a Shiseido MG-C18 column (3.0 × 100 mm, i.d.; 3.0 μm, Tokyo, Japan) at room temperature. The mobile phase was water (containing 0.1% formic acid) and acetonitrile (36:64, v:v) at a flow rate of 0.6 mL·min^−1^, and the split ratio was 1:1.

The mass scan mode was positive MRM mode. The precursor ion and product ion are *m/z* 361.3→128.2 for triptolide, and *m/z* 363.5→121.0 for the IS, respectively. The collision energy for triptolide and IS were 30 and 20 ev, respectively. The MS/MS conditions were optimized as follows: fragmentor, 110 V; capillary voltage, 3.5 kV; Nozzle voltage, 500 V; nebulizer gas pressure (N_2_), 40 psig; drying gas flow (N_2_), 10 L·min^−1^; gas temperature, 350 °C; sheath gas temperature, 400 °C; sheath gas flow, 11 L·min^−1^.

### 3.3. Pharmacokinetic Study in Vivo

#### 3.3.1. Animals

Male Sprague-Dawley (SD) rats weighing 180-220 g were provided by the Experimental Animal Center of the Guangdong Pharmaceutical Medical University (Guangdong, China). Rats were bred in a breeding room at 25 °C, 60% ± 5% humidity, and a 12 h dark-light cycle. Tap water and normal chow were given ad libitum. All the experimental animals were housed under the above conditions for 5-day acclimation, and fasted overnight before the experiments.

#### 3.3.2. *In Vivo* Pharmacokinetic Study

The animal facilities and protocols were approved by the Institutional Animal Care and Use Committee. All procedures were in accordance with the National Institute of Health guidelines regarding the principles of animal care. Rats were fasted for 12 h with free access to water prior to the pharmacokinetic study. Twelve rats were equally randomized to two groups and were treated as followings: intravenous injection of triptolide in saline was administrated through lateral tail vein at dose of 1 mg·kg^−1^. An oral gavage of triptolide dispersed in oral suspension vehicle was given to rats at the same dose. Blood samples (200 μL) were collected into a heparinized tube via the oculi chorioideae vein at 0, 2, 5, 10, 15, 30, 45, 60, 90, 120, 180, 240, and 360 min, respectively. After centrifugation at 4000 rpm for 10 min, plasma samples were obtained and frozen at −40 °C until analysis. 

#### 3.3.3. Plasma Sample Preparation

The protein precipitation method in 96 well format was used for preparing the samples. Aliquots of plasma samples (50 μL) were pipetted into 96 well plates. IS (hydrocortisone, 10 μL, 1 μg·mL^−1^) and acetonitrile (90 μL) were added to the samples to precipitate the protein. Plates were capped and mixed and vortexed for 3 min, then the plates were centrifuged at 5000 rpm for 15 min at 4 °C to remove any precipitated material. A sample (100 μL) of the supernatant was transferred to another Sirocco 96 well filtration plate and then placed in the 96 well manifold, allowing plasma to be filtered by vacuum for 5 min. An aliquot of 5 μL of the filtrate was injected into the LC-MS/MS system for quantitative analysis.

#### 3.3.4. Preparation of Standard and Quality Control Samples

A stock solution of triptolide was prepared in acetonitrile at a concentration of 2 mg·mL^−1^. The stock solution of IS was prepared in acetonitrile at a concentration of 1 μg·mL^−1^. Calibration standard samples for triptolide were prepared in blank rat plasma at concentrations of 5, 10, 25, 50, 100, 500, 1000 ng·mL^−1^. The quality control (QC) samples were prepared at low (10 ng·mL^−1^), medium (50 ng·mL^−1^), and high (500 ng·mL^−1^) concentrations in the same way as the plasma samples for calibration, and QC samples were stored at −40 °C until analysis.

#### 3.3.5. Method Validation

The method validation assay was performed according to the United States Food and Drug Administration (FDA) guidelines. Selectivity was investigated by comparing the chromatograms of six different batches of blank rat plasma with the corresponding spiked plasma to monitor interference of endogenous substances and metabolites. To obtain the calibration curve, seven concentrations of the calibration standard were processed and determined as described above. The linearity of calibration curves was constructed by plotting peak area ratios (y) of the analytes to IS against the nominal concentration (x) of analytes with weighted (1/x^2^) least square linear regression. The lower limit of detection (LLOD) and lower limit of quantification (LLOQ) were determined as the concentration of the analyte with a signal-to-noise ratio at 3 and 10, respectively. The intra-day precision and accuracy of the method were confirmed by determining QC samples at three different concentrations five times on a single day, and the inter-day precision and accuracy were assessed by determining the QC samples over three consecutive days. For each concentration, five replicates were prepared. Relative standard deviation (RSD) and relative error (RE) were used to express the precision and accuracy, respectively.

The extraction recovery was assessed by comparing peak areas obtained from extracted spiked samples with those originally spiked in the blank plasma samples (n = 5). The matrix effect was evaluated by comparing the peak areas of the post-extracted spiked QC samples with those of corresponding standard solutions. These procedures were repeated for five replicates at three QC concentration levels. For sample stability, three levels of QC samples were determined under different conditions, including short-term stability at room temperature for 24 h, long-term stability at −40 °C for 30 days, and three freeze-thaw cycles at −40 °C.

#### 3.3.6. Data Analysis

The pharmacokinetic parameters, including area under the plasma concentration-time curve (*AUC*), maximal plasma concentration (*C_max_*), the time for maximal plasma concentration (*T_max_*), and mean residence time (*MRT*) were calculated using DAS 3.0 pharmacokinetic software (Chinese Pharmacological Association, Anhui, China).

### 3.4. Caco-2 Cell Monolayer Model

#### 3.4.1. Cell Culture

Caco-2 cell line was obtained from the American Type Culture Collection (Manassas, VA, USA). Caco-2 cell transport experiment was performed according to the previously reported method [18,19]. In brief, Caco-2 cells were cultured in DMEM high glucose media containing 15% FBS, 1% NEAA and 100 U·mL^−1^ penicillin and streptomycin. The cells were cultured at 37 °C with 5% CO_2_. For transport studies, cells at passage 30-40 were seeded on transwell polycarbonate insert filters (1.12 cm^2^ surface, 0.4 μm pore size, 12 mm diameter; Corning Costar Corporation, New York, MA, USA) in 12-well plates at a density of 1 × 10^5^ cells·cm^−2^. Cells were allowed to grow for 21 days. For the first seven days, the media was replaced every two days, and it was replaced daily thereafter. The transepithelial electrical resistance (TEER) of monolayer cells was measured using Millicell ERS-2 (Millipore Corporation, Billerica, MA, USA), and TEER value should exceded 400 Ω·cm^2^. The integrity of the Caco-2 monolayers was confirmed by the paracellular flux of Lucifer yellow which was less than 1% per hour. The alkaline phosphatase activity was validated using an Alkaline Phosphatase Assay Kit. The qualified monolayers were used for transport studies.

#### 3.4.2. Transport Studies

Before the transport experiments, cell monolayers were rinsed twice using warm (37 °C) Hanks’ balanced salt solution (HBSS), and then the cell were incubated at 37 °C for 20 min. After preincubation, cell monolayers were incubated with triptolide in fresh incubation media from either the apical or basolateral side for the designated times at 37 °C. The volume of incubation media on the apical and basolateral sides was 0.5 mL and 1.5 mL, respectively, and a 100 μL aliquot of the incubation solution was withdrawn at the designated time points from the receiver compartment and replaced with the same volume of fresh pre-warmed HBSS buffer. The inhibitory effects of *P-gp* inhibitors on triptolide flux by Caco-2 cells were investigated by adding 50 μM verapamil to both sides of the cell monolayers. The permeability of triptolide (10 μM) in all the above conditions for both directions, from the apical (AP) to basolateral (BL) side or from the BL to AP side, was measured after incubation for 30, 60, 90 and 120 min at 37 °C. The Caco-2 cell transport media sample preparation method was the same as the plasma sample preparation method.

#### 3.4.3. Data Analysis in the Caco-2 Cell Monolayer Model

The apparent permeability coefficients (*P_app_*) was calculated using the equation of Artursson and Karlsson: *P_app_* = (ΔQ/Δt) × [1/(A × C_0_)], where *P_app_* is the apparent permeability coefficient (cm·s^−1^), ΔQ/Δt (μmol·s^−1^) is the rate at which the compound appears at the receiver chamber, C_0_ (μmol·L^−1^) is the initial concentration of the compound in the donor chamber and A (cm^2^) represents the surface area of the cell monolayer. Data were collected from the three separate experiments, and each was performed in triplicate.

### 3.5. Metabolic Stability in Human Liver Microsomes

The rat liver microsome incubation experiment was performed according to the previously reported method [20]. In brief, NADPH-generating system (10 mM G-6-P, 1 mM NADP^+^, 4 mM magnesium chloride, 1 unit·mL^−1^ of G-6-PDH), 10 μL human liver microsome (20 mg·mL^−1^), 4 μL drug solution (100 μM) and 366 μL PBS buffer (0.1 M, *pH* 7.4) were added to centrifuge tubes stored on ice. The sample at t = 0 was achieved by removing a sample of incubation mixture (95 μL), adding PBS buffer (5 μL) and mixing with acetonitrile (300 μL). There was a 3 min preincubation step at 37 °C before initiating the reaction by adding NADPH-generating system into the microsomal suspension. A 30 μL reaction sample was collected at 5, 10, 15, 20, 30, 45 and 60 min, and then 60 μL of ice cold acetonitrile containing 50 ng/mL IS was added to terminate the reaction, and then the human liver microsome incubation media sample preparation method was the same as the plasma sample preparation method. All depletion data were fitted to the monoexponential decay model described by the equation: *C(t) = C_0_e^−kt^.* The *in vitro* half-life (*T_1/2_*) was obtained using the equation: *T_1/2_ = −0.693/k*.

## 4. Conclusions

In this study, a rapid and specific LC-MS/MS method has been developed and successfully applied to analyzing triptolide in plasma, Caco-2 transport media, and microsome samples. The precision, accuracy, extraction recovery, and stability of this method were validated. Using this method, the pharmacokinetic characteristics of triptolide in rats, the transport mechanism, and metabolism stability were systematically investigated. The results indicated that *P-gp* might be involved in the absorption of triptolide, and it was easily metabolized in human liver microsomes. 
